# Migration-inducing gene 7 promotes tumorigenesis and angiogenesis and independently predicts poor prognosis of epithelial ovarian cancer

**DOI:** 10.18632/oncotarget.8487

**Published:** 2016-03-30

**Authors:** Bihui Huang, Mingzhu Yin, Xia Li, Guosheng Cao, Jin Qi, Ge Lou, Shijie Sheng, Junping Kou, Kang Chen, Boyang Yu

**Affiliations:** ^1^ Department of Pharmacology, Yale University School of Medicine, New Haven, Connecticut, USA; ^2^ Department of Obstetrics and Gynecology, Wayne State University, Detroit, Michigan, USA; ^3^ Perinatology Research Branch, Eunice Kennedy Shriver National Institute of Child Health and Human Development, National Institutes of Health, Detroit, Michigan, USA; ^4^ State Key Laboratory of Natural Products and Jiangsu Key Laboratory of Traditional Chinese Medicine (TCM) Evaluation and Translational Research, Department of Complex Prescription of TCM, China Pharmaceutical University, Nanjing, China; ^5^ Department of Pathology, Yale University School of Medicine, New Haven, Connecticut, USA; ^6^ Department of Internal Medicine, Yale University School of Medicine, New Haven, Connecticut, USA; ^7^ Yale Stem Cell Center, Yale University, New Haven, Connecticut, USA; ^8^ Department of Gynecologic Oncology, The Affiliated Cancer Hospital of Harbin Medical University, Harbin, China; ^9^ Department of Pathology, Wayne State University, Detroit, Michigan, USA; ^10^ Tumor Biology and Microenvironment Program, Barbara Ann Karmanos Cancer Center and Department of Oncology, Wayne State University, Detroit, Michigan, USA; ^11^ Department of Immunology and Microbiology, Wayne State University, Detroit, Michigan, USA; ^12^ Mucosal Immunology Studies Team, National Institute of Allergy and Infectious Diseases, National Institutes of Health, Bethesda, Maryland, USA

**Keywords:** epithelial ovarian cancer, biomarker, prognosis, Migration-Inducting Gene 7, angiogenesis

## Abstract

Epithelial ovarian carcinomas (EOC) cause more mortality than any other cancer of the female reproductive system. New therapeutic approaches to reduce EOC mortality have been largely unsuccessful due to the poor understanding of the mechanisms underlying EOC proliferation and metastasis. Progress in EOC treatment is further hampered by a lack of reliable prognostic biomarkers for early risk assessment. In this study, we identify that Migration-Inducting Gene 7 (MIG-7) is specifically induced in human EOC tissues but not normal ovaries or ovarian cyst. Ovarian MIG-7 expression strongly correlated with EOC progression. Elevated MIG-7 level at the time of primary cytoreductive surgery was a strong and independent predictor of poor survival of EOC patients. Cell and murine xenograft models showed that MIG-7 was required for EOC proliferation and invasion, and MIG-7 enhanced EOC-associated angiogenesis by promoting the expression of vascular endothelial growth factor. Inhibiting MIG-7 by RNA interference in grafted EOC cells retarded tumor growth, angiogenesis and improved host survival, and suppressing MIG-7 expression with a small molecule inhibitor D-39 identified from the medicinal plant *Liriope muscari* mitigated EOC growth and invasion and specifically abrogated the expression of vascular endothelial growth factor. Our data not only reveal a critical function of MIG-7 in EOC growth and metastasis and support MIG-7 as an independent prognostic biomarker for EOC, but also demonstrate that therapeutic targeting of MIG-7 is likely beneficial in the treatment of EOC.

## INTRODUCTION

Ovarian cancer is one of the most common gynecologic malignancies and causes more mortality than any other cancer of the female reproductive system [1]. Nearly 90% of ovarian cancer is classified as epithelial ovarian carcinomas (EOC) [1]. Approximately 70% of EOC patients are in the advanced stage at the time of diagnosis with incurable disease [2–4]. Despite advances in sophisticated surgery and new chemotherapeutic regimens, the 5-year survival rate for EOC patients has not achieved much improvement in the last two decades [5]. Failure of effective EOC treatment has been largely due to a lack of reliable biomarkers for early risk assessment [6] and uncontrolled cancer cell proliferation, metastasis and the acquisition of chemoresistance in the advanced stage [7]. EOC cells have high tolerance to low oxygen, which enables them to evade chemotherapy and rapidly develop drug resistance [8]. To support the rapid growth, it is necessary for EOC cells to acquire sufficient nutrients via promoting tumor angiogenesis [9]. Therefore, understanding the regulators of EOC proliferation and angiogenesis will reveal novel molecular targets for drugs to inhibit these critical processes, and identifying reliable prognostic biomarkers will help to improve risk assessment, disease management and consequently patient survival.

Migration-Inducting Gene 7 (MIG-7) is a cysteine-rich protein first identified in endometrial cancer cells upon hepatocyte growth factor (HGF) treatment [10]. Later studies showed that MIG-7 was upregulated on cell membrane and in the cytoplasm of a variety of cancers, but remained low or undetectable in non-cancerous tissues [11]. MIG-7 may therefore represent a promising marker for cancer detection and diagnosis. This notion is further supported by immunohistochemical (IHC) studies showing MIG-7 expression in circulating tumor cells, suggesting its potential as an early marker for metastatic carcinomas [11]. Studies on the function of MIG-7 in regulating tumor growth were focused primarily on two aspects. Some suggested that MIG-7 promoted tumor growth by regulating mitogenic and metastatic signaling pathways [12]. For example, MIG-7 could promote metastasis of lung cancer by activating the cyclooxygenase-2 (COX-2)–prostaglandin E2 (PGE2) signaling cascade [13]. Others suggested that MIG-7 promoted vascular mimicry prior to tumor angiogenesis, thus contributing to early EOC growth and metastasis [14]. However, currently there are no effective drugs that specifically inhibit vascular mimicry to treat EOC. Nor is there any report on the level of MIG-7 expression on the prognosis of EOC.

In the present study, we followed a cohort of EOC patients and found that elevated ovarian MIG-7 expression strongly correlated with EOC progression and metastasis, and independently predicted poor prognosis. We further showed that MIG-7 promotes EOC growth and angiogenesis *in vitro* and *in vivo*, and may represent an effective therapeutic target of EOC.

## RESULTS

### Elevated ovarian MIG-7 expression correlates with EOC progression and metastasis

**Figure 1:**
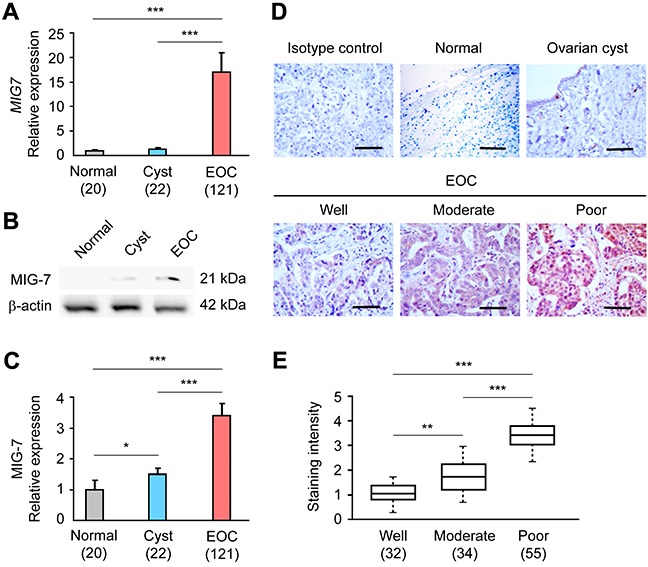
MIG-7 expression is elevated in EOC and correlates with advanced disease **A.** qRT-PCR analysis of *MIG7* transcriptional level in normal ovary, ovarian cyst and EOC tissues. The relative expression of the *MIG7* gene is normalized to the expression of the *ACTB* (β-actin) gene. **B, C.** Western Blot and densitometric analyses of MIG-7 expression in normal ovary, ovarian cyst and EOC tissues. β-actin served as a loading control and was used for normalization. **D.** Representative IHC staining images of MIG-7 in normal ovary, ovarian cyst and EOC tissues at different FIGO stages. Bar: 100 μm. **E.** Statistical analysis of MIG-7 staining intensity in well, moderately and poorly differentiated EOC samples. The staining was scored by positivity and intensity. All data are presented as Mean ± SEM. The sample size of each group is indicated in the parentheses.*: *p* < 0.05; **: *p* < 0.01; ***: *p* < 0.001.

The function of MIG-7 as a cancer-promoting factor and a biomarker in EOC has been unknown. Using quantitative real-time polymerase chain reaction (qRT-PCR) and Western Blot analyses, we found that MIG-7 mRNA and protein levels were increased in the ovaries of a cohort of 121 EOC patients as compared to subjects with normal ovaries or patients with ovarian cyst (Figure 1A–1C). Of note, MIG-7 protein was undetectable in normal ovarian tissues. IHC analysis confirmed increased ovarian MIG-7 protein expression in EOC (Figure 1D). MIG-7 expression in EOC tissues correlated positively with tumor stage (*p* = 0.0008) and negatively with histopathological differentiation (*p* = 0.0001) (Figure 1E and Table 1). Furthermore, EOC patients with high ovarian MIG-7 expression had significantly more ascites volume and lymph node metastasis than those with low ovarian MIG-7 expression (*p* = 0.009 and 0.03, respectively) (Table 1). No statistically significant association between MIG-7 expression and other clinicopathological parameters, such as serum CA-125 level and histology type, was found. Therefore, elevated ovarian MIG-7 expression closely associates with the progression, de-differentiation and metastasis of EOC.

**Table 1:** Association between MIG-7 expression and clinicopathological features of EOC

Clinicopathological parameter	MIG-7 expression		No. of patients (*n* = 121)	*p*
	LOW	HIGH		
**Age (years)**				0.32
<50	18	25	43	
≥ 50	26	52	78	
**Serum CA-125 level (U/ml)**				0.48
< 35	4	6	10	
≥ 35	40	71	111	
**Ascites (ml)**				0.009
< 100	34	5	39	
≥ 100	10	72	82	
**Histopathological differentiation**				0.0001
Well	25	7	32	
Moderate	17	17	34	
Poor	2	53	55	
**Histology type**				0.412
Serous adenocarcinoma	33	48	81	
Mucoid adenocarcinoma	5	7	12	
Endometrioid adenocarcinoma	6	22	28	
**FIGO stage**				0.0008
I & II	32	10	42	
III & IV	12	67	79	
**Lymph node metastasis**				0.03
Absent	9	11	20	
Present	35	66	101	
**Primary tumor size (cm)**				0.63
< 5	14	27	41	
≥ 5	30	50	80	

### MIG-7 is required for EOC proliferation and invasion

**Figure 2:**
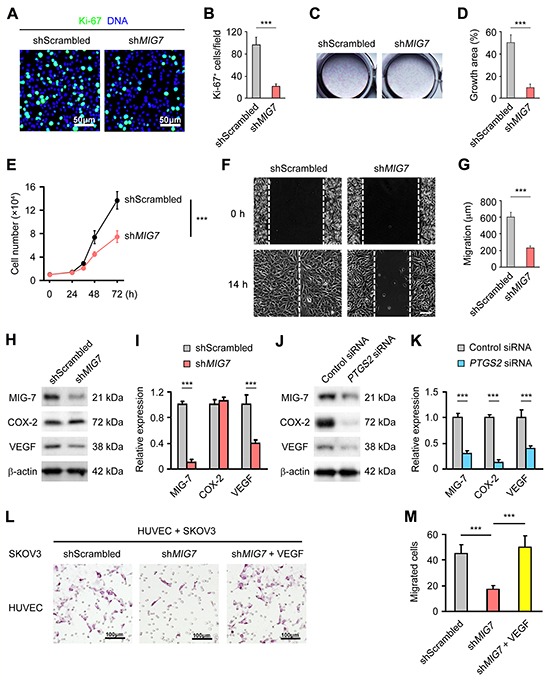
MIG-7 promotes EOC cell proliferation, invasion and angiogenesis in vitro **A, B.** Immunofluorescent images and statistical analyses of Ki-67 expression in SKOV3 cells with control or *MIG7* stable knockdown by scrambled or *MIG7*-targeting shRNA, respectively. Bar: 50 μm. **C, D.** Clonogenic assay of SKOV3 cells following control or *MIG7* stable knockdown. **E.** Proliferation of SKOV3 cells with control or *MIG7* stable knockdown in culture for 24, 36, 48 and 72 h. 1×10^4^ cells were seeded at the beginning of the assay. **F, G.** Invasion of SKOV3 cells with control or *MIG7* stable knockdown determined by a wound healing assay. White dashed lines indicate the gap of the cell monolayer at 0 and 14 after cell scratch. Bar: 200 mm. **H, I.** Western Blot and densitometric analyses of COX-2, MIG-7 and VEGFA expression in SKOV3 cells with control or *MIG7* stable knockdown. β-actin served as a loading control. **J, K.** Western Blot and densitometric analyses of COX-2, MIG-7 and VEGFA expression in SKOV3 cells with control or *PTGS2* (COX-2) knockdown by siRNA. β-actin served as a loading control. **L, M.** Hematoxylin staining and statistical analysis of HUVEC transmigration after 14 h of co-culture with SKOV3 cells with control or *MIG7* stable knockdown with or without 100 ng/ml VEGFA. Each experiment was performed in triplicates. Data are presented as mean ± SEM of triplicates and represent 3 independent experiments. ***: *p* < 0.001.

To understand the function of MIG-7 expression in EOC development, we screened a panel of gynecological epithelial cancer cell lines by qRT-PCR for *MIG7* expression. *MIG7* was highly expressed in the EOC line SKOV3 (Supplementary Figure S1). Stable knockdown of *MIG7* (Supplementary Figure S2A and S2B) resulted in a significant reduction in the proliferation of SKOV3 cells, as evidenced by diminished numbers of Ki-67^+^ cells (Figure 2A and 2B) [15], impaired ability to form colonies (Figure 2C and 2D) and reduced growth (Figure 2E). In addition, MIG-7 knockdown markedly blunted the invasiveness of SKOV3 cells, as shown by their reduced migration in a wound healing assay (Figure 2F and 2G). These data suggest that MIG-7 is required for the proliferation and invasiveness of EOC cells.

### MIG-7 promotes VEGF expression by EOC cells

Tumor-associated angiogenesis promotes tumor growth and metastasis [16]. Both mRNA and protein levels of vascular endothelial growth factor A (VEGFA), a major angiogenic factor, were significantly reduced in SKOV3 cells upon *MIG7* knockdown (Supplementary Figure S3A and Figure 2H and 2I). Of note, MIG-7 knockdown did not affect the expression of COX-2 (Figure 2H and 2I), while knocking down COX-2 expression diminished the level of both MIG-7 and VEGFA (Supplementary Figure S3B and Figure 2J and 2K), which was consistent with COX-2 being an upstream inducer of MIG-7 [13]. Accordingly, SKOV3 cells with MIG-7 expression knocked down had impaired ability to induce the migration of co-cultured HUVECs, which was rescued by the addition of exogenous VEGFA (Figure 2L and 2M). Indeed, we found a significant positive correlation between the expression of MIG-7 and that of VEGFA in primary EOC tissues (Correlation Index (CI) = 0.37; *p* < 0.0001).

### MIG-7 promotes EOC growth and angiogenesis and impairs host survival

**Figure 3:**
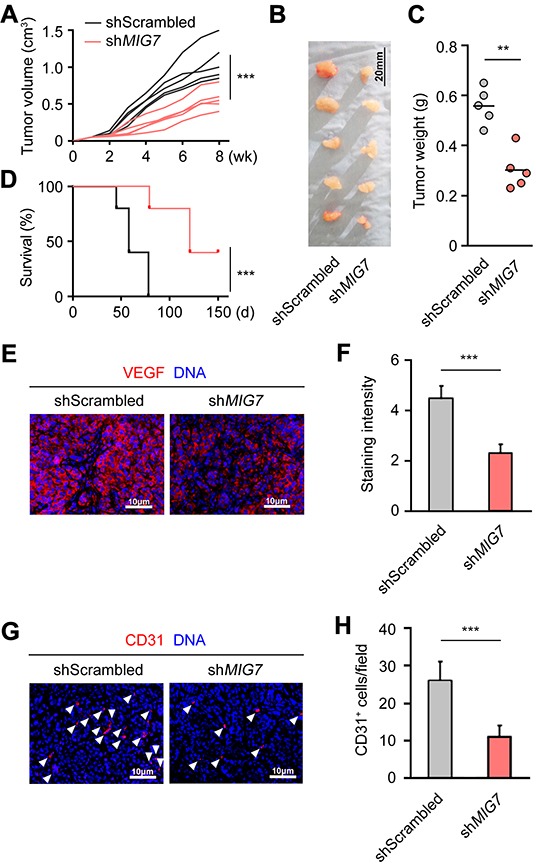
MIG-7 is required for the growth and angiogenesis of EOC cells in vivo **A.** Volume of SKOV3-drived control or *MIG7*-knockdown tumors after inoculation, measured every week for 8 weeks. **B.** Images of SKOV3-derived control or *MIG7*-knockdown tumors 60 days after subcutaneous inoculation of SKOV3 cells into athymic nude mice. **C.** Weight of SKOV3-derived control or *MIG7*-knockdown tumors 60 days after inoculation. Bar: 20 mm; *n* = 5. **D.** Kaplan-Meier analysis of the survival of athymic nude mice inoculated with 10^7^ control or *MIG7*-knockdown SKOV3 cells. **E, F.** Immunofluorescent images and statistical analysis of VEGFA (red) expression in dissected tumors from athymic nude mice 60 days after subcutaneous inoculation of control or *MIG7*-knockdown SKOV3 cells. DAPI stains the DNA (blue). Bar: 10 mm. **G, H.** Immunofluorescent images and statistical analysis of CD31 (red, and indicated by arrowheads) expression in dissected tumors from athymic nude mice 60 days after subcutaneous inoculation of control or *MIG7*-knockdown SKOV3 cells. DAPI stains the DNA (blue). Bar: 10 mm. Each experiment was performed with 5 mice per group. Data are presented as mean ± SEM of 5 mice and represent 3 independent experiments. **: *p* < 0.01; ***: *p* < 0.001.

To determine the function of MIG-7 on EOC growth *in vivo*, we inoculated SKOV3 cells with control or *MIG7* stable knockdown into athymic nude mice. Tumors derived from *MIG7* knockdown cells exhibited markedly attenuated growth (Figure 3A) and reduced size than control tumors (Figure 3B and 3C). Mice inoculated with *MIG7* knockdown tumor cells experienced delayed death and improved rate of survival (Figure 3D). Consistent with the growth retardation of *MIG7* knockdown tumors, cells in these tumors exhibited diminished expression of the proliferation-associated molecule Ki-67 (Supplementary Figure S4). Furthermore, VEGFA expression and tumor angiogenesis were markedly reduced in *MIG7* knockdown tumors (Figure 3E–3H), echoing the result found in SKOV3 cells *in vitro*. Taken together, these data show that MIG-7 is an important promoting factor of EOC pathogenesis, including tumor proliferation, migration and angiogenesis.

### A small molecule inhibitor of MIG-7 expression suppresses EOC growth, invasion and angiogenesis

**Figure 4:**
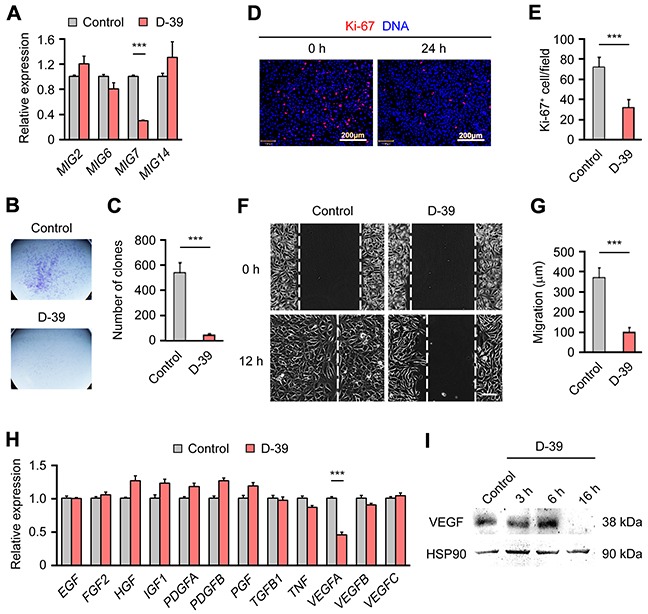
D-39, an inhibitor of MIG-7 expression, suppresses EOC cell growth and VEGFA expression **A.** qRT-PCR analysis of *MIG2*, *MIG6*, *MIG7* and *MIG14* gene expression in SKOV3 cells treated with 20 μM D-39 for 14 h. Values were normalized to cells treated with 2% dimethyl sulfoxide (DMSO) (control), which was the solvent present in 20 μM D-39. **B, C.** Growth of SKOV3 cells treated with 2% DMSO (control) or 20 μM D-39 for 14 h, as determined by a clone formation assay. **D, E.** Immunofluorescent images and statistical analyses of Ki-67 expression in SKOV3 cells treated with 2% DMSO (control) or 20 μM D-39 for 14 h. DAPI stained DNA. Bar: 200 μm. **F, G.** Invasion of SKOV3 cells treated with 2% DMSO (control) or 20 μM D-39 for 14 h, as determined by a wound healing assay. White dashed lines indicate the gap between the cell monolayer at 0 and 12h after the scratch. **H.** qRT-PCR analysis of Epidermal growth factor (*EGF*), Fibroblast growth factor 2 (*FGF2*), Hepatocyte growth factor (*HGF*), Insulin-like growth factor 1 (*IGF1*), Platelet-derived growth factor β (*PDGFA*), Platelet-derived growth factor β (*PDGFB*), Placental growth factor (*PGF*), Transforming growth factor-β (*TGFB1*), Tumor necrosis factor (*TNF*), *VEGFA*, *VEGFB* and *VEGFC* gene expression in SKOV3 cells treated with 20 mM D-39 for 14 h. Values were normalized to the cells treated with 2% DMSO (control). **I.** Western Blot analysis of VEGFA expression in SKOV3 cells treated with 2% DMSO (control) or 20 μM D-39 for 3, 6 and 16 h. HSP90 served as a loading control. Each experiment was done in triplicates. Data are presented as mean ± SEM of triplicates and represent 2 to 4 independent experiments. Bar: 200μm; ***: *p* < 0.001.

Having demonstrated a critical function of MIG-7 in EOC pathogenesis, we explored the therapeutic potential of inhibiting MIG-7 in EOC. To this end, we performed a screen for inhibitors of MIG-7 expression from the compound library of *Liriope muscari*, a medicinal plant used in traditional Chinese medicine to treat various diseases including malignancies [17]. A compound, D-39 (Supplementary Figure S5A), was identified, which specifically suppressed the expression of *MIG7*, but not *MIG2*, *MIG6* or *MIG14 in SKOV3 cells* (Figure 4A). Treatment of D-39 did not decrease the expression of COX-2, an inducer of MIG-7 (data not shown) [13]. D-39 exhibited selective killing of SKOV3 cells but not A2780 cells which have an undetectable level of MIG-7 (Supplementary Figure S5B). Consistent with a positive role of MIG-7 in EOC growth and invasion, D-39 potently inhibited the growth, proliferation and invasion of SKOV3 cells (Figure 4B–4G). Our earlier results demonstrated a requirement for MIG-7 in VEGF expression in EOC. Accordingly, the MIG-7 inhibitor D-39 specially suppressed the expression of VEGFA but not other angiogenic or mitogenic factors (Figure 4H and 4I). Collectively, our findings suggest that therapeutic targeting MIG-7 may be beneficial in inhibiting multiple key processes of EOC pathogenesis, including growth, angiogenesis and metastasis (Supplementary Figure S7).

### MIG-7 expression in EOC tissues positively correlates with EOC angiogenesis

**Figure 5:**
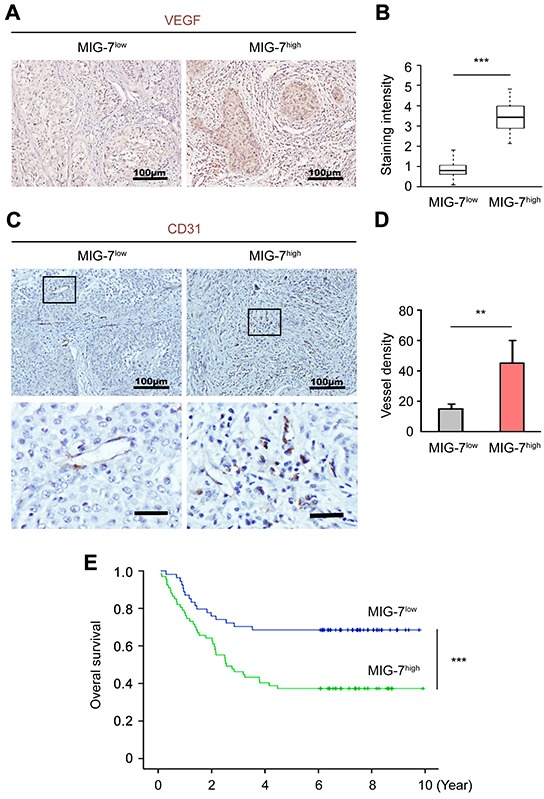
High ovarian MIG-7 expression independently predicts poor prognosis of EOC **A, B.** Immunohistochemical staining and statistical analysis of VEGFA expression in resected EOC samples with low or high MIG-7 expression. Nuclei were stained with hematoxylin. Bar: 100 μm. **C, D.** IHC and statistical analyses of CD31 expression in resected EOC samples with low or high MIG-7 expression. Nuclei were stained with hematoxylin. Bar: 100 mm (upper panels), 25 mm (lower panels). **E.** Kaplan-Meier analysis of the OS of EOC patients with either high or low expression of MIG-7 at the time of primary cytoreductive surgery. The median survival of EOC patients with high and low MIG-7 expression is 3.8 and 5.6 years, respectively. **: *p* < 0.01; ***: *p* < 0.001. *n* = 44 (MIG-7^low^) and 77 (MIG-7^high^).

To confirm the correlation between MIG-7 expression and angiogenesis in human EOC, we determined tumor-associated angiogenesis in EOC tissues of 52 and 69 patients with low and high MIG-7 expression, respectively. VEGFA expression was significantly elevated in patients with high MIG-7 expression, particularly in the ovary area containing aggregated tumor cells (Figure 5A and 5B). Poisson analysis revealed a significant positive correlation between MIG-7 and VEGFA expression (CI = 0.37, *p* < 0.0001). Consistently, tumors with high MIG-7 expression harbored a higher density of tumor-associated vessels as determined by CD31 staining (Figure 5C and 5D). Therefore, MIG-7 expression in EOC tissues positively correlates with EOC-associated angiogenesis.

### Ovarian MIG-7 expression independently predicts poor prognosis of EOC

We further determined the prognostic value of ovarian MIG-7 expression in patient survival. Univariate analysis from 121 EOC patients with either low (*n* = 52) and high (*n* = 69) MIG-7 expression revealed that the 5-year OS for patients with high and low tumor MIG-7 expression was 39.39% and 65.15%, respectively (*p* < 0.0001) (Figure 5E and Table 2). Further analysis suggested that higher MIG-7 expression negatively correlated with the OS of EOC patients (*p* = 0.0008), alongside classical clinicopathological variables including residual tumor size (*p* = 0.02), histopathological differentiation (*p* = 0.0161), lymph node metastasis (*p* = 0.0001), CA-125 level (*p* = 0.0321), ascites volume (*p* = 0.0352), FIGO stage (*p* = 0.0041) and lymph node metastasis (*p* < 0.0001) (Table 2). A multivariate Cox regression model adjusted for statistically significant prognostic factors was further established to evaluate the independent impact of MIG-7 expression on the OS. In this model, OS was used as a response variable and the factors used for adjustment included residual tumor size, CA-125 level, ascites volume, lymph node metastasis, histopathological differentiation and FIGO stage. Patients with high MIG-7 expression had a significantly shorter OS than those with low MIG-7 expression (OS: Hazard ratio = 2.099, *p* = 0.0133) (Table 3). In conclusion, out data suggest that MIG-7 is a novel and powerful biomarker that independently predicts poor prognosis of EOC.

**Table 2:** Univariate analysis of 113 EOC patients

Variable	OS		*p*
	3-year	5-year	
**MIG-7 expression**			0.0008
Low	0.7455	0.6515	
High	0.6909	0.3939	
**Residual tumor size (cm)**			0.02
< 1	0.8872	0.8455	
≥ 2	0.5909	0.4545	
**FIGO stage**			0.0041
I & II	0.8636	0.8182	
III & IV	0.6566	0.4646	
**Serum CA-125 level (U/ml)**			0.0321
< 35	0.9167	0.8333	
≥ 35	0.6697	0.4954	
**Ascites (ml)**			0.0352
< 100	0.8276	0.6897	
≥ 100	0.6522	0.4783	
**Lymph node metastasis**			< 0.0001
Absent	0.7426	0.604	
Present	0.45	0.15	
**Histopathological differentiation**			0.0161
G1	0.4314	0.6275	
G2	0.175	0.375	
G3	0.7333	0.6667	
**Histology type**			0.6894
Serous adenocarcinoma	0.6951	0.5244	
Mucoid adenocarcinoma	0.6875	0.625	
Endometrioid adenocarcinoma	0.65	0.45	

**Table 3:** Multivariate analysis with covariates adjustment of 121 EOC patients

Variable	Parameter estimate	*p*	Hazard ratio	95% Hazard Ratio	Confidence Limits
**MIG-7 expression**					
Low					
High	0.74139	0.0133	2.099	1.167	3.776
**FIGO stage**					
I & II					
III & IV	0.57696	0.3044	1.781	0.592	5.355
**Serum CA-125 level (U/ml)**					
< 35					
≥ 35	0.95485	0.1938	2.598	0.616	10.968
**Ascites (ml)**					
<100					
≥ 100	0.55200	0.1618	1.737	0.801	3.764
**Lymph node metastasis**					
Absent					
Present	0.96390	0.0018	2.622	1.433	4.797
**Histopathological differentiation**					
G1					
G2	−0.58562	0.0551	0.557	0.306	1.013
G3	−0.32701	0.3971	0.721	0.338	1.537

## MATERIALS AND METHODS

### Cell lines

Human embryonic kidney epithelial cell line HEK293T, breast cancer cell line MCF-7, trophoblast cell line HTR-8/svNeo, endometrial cancer cell Ishikawa and three lines of ovarian cancer cells IGROV1, A2780 and SKOV3 were purchased from American Type Culture Collection (ATCC, Manassas, VA, USA). All cells were cultured in Dulbecco's Modified Eagle Medium (Thermo Fisher Scientific, Carlsbad, CA, USA) supplemented with 10% fetal bovine serum (Clontech Laboratories, Mountain View, CA, USA), 10 U/ml penicillin and 10 mg/ml streptomycin in a humidified incubator at 37°C with 5% CO_2_.

### Study subjects

Following Institutional Review Board approval, a total of 121 patients with advanced EOC seen at the Affiliated Cancer Hospital of Harbin Medical University from January 2003 to December 2011 who met the following inclusion criteria were enrolled into this study. The inclusion criteria were: (1) pathology examination confirming the presence of stage I to IV EOC; (2) complete basic clinical data; (3) no prior treatment for cancer; (4) absence of serious complications or other malignant disease; (5) the patients and family members being informed about the illness and having given informed consent before treatment. All patients had undergone complete cytoreductive surgery. After obtaining informed consent, all eligible patients received intravenous platinum-based combination chemotherapy after surgery consisting of 6 cycles of treatment with 3 weeks between each cycle. An additional 42 subjects with pathologically confirmed normal ovaries or ovarian cysts were included as the control.

### RNA interference

To achieve stable knockdown of *MIG7*, SKOV3 cells were transfected at 70% confluence with 4 mg *MIG7*-targeting shRNA and selected with 2 mg/ml puromycin. For transient knockdown, SKOV3 cells were transfected with 40 nM *MIG7*-*or PTGS2*-targeting siRNA (GE Dharmacon, Lafayette, CO, USA) and harvested at 72 h for mRNA and protein extraction. The sequences of the shRNA and siRNA used are provided in Supplementary Table S1 [13, 18].

### qRT-PCR

Total RNA was extracted from cells and tissues using an RNAeasy Kit (Qiagen, Redwood City, CA, USA). 500 ng of RNA was reverse transcribed into cDNA using an iScript reverse transcription kit (Bio-Rad, Hercules, CA, USA). Equal volume of cDNA was applied for qRT-PCR using SYBR Green (Bio-Rad) with the following thermocycling condition: 15 seconds (s) at 95°C and 1 min at 60°C for 40 cycles, and the primers in Supplementary Table S2.

### Western blot

Western Blot analysis was performed with tissue samples homogenized in lysis buffer consisting of 1% Triton X-100 and a protease inhibitor cocktail in phosphate-buffered saline (PBS). The lysates were centrifuged at 12 000 g for 15 min at 4°C to obtain the supernatants. Sixty micrograms of the protein extract was separated by 10% SDS polyacrylamide gel electrophoresis (SDS-PAGE) and transferred onto a PVDF membrane (EMD Millipore, Billerica, MA, USA). The membrane were blocked and incubated with antibodies against target proteins. Immunoreactive proteins were revealed by chemiluminescence. Membranes were then stripped and treated as described above but with an antibody against β-actin. Antibodies used are listed in Supplementary Table S3.

### Immunohistochemistry

MIG-7 expression was evaluated in ovarian tissues of 121 EOC patients, 22 patients with ovarian cysts and 20 subjects with normal ovaries using the avidin-biotin immunoperoxidase technique. 4 mm paraffin-embedded tissue sections were stained with hematoxylin and eosin for tumor confirmation. Adjacent sections were used for IHC analysis. The selected sections were deparaffinized in Xylene and rehydrated with graded alcohol, immersed in 0.01 M citrate buffer (pH = 6.0) and heated at 95°C for 15 minutes (min). Sections were incubated with 3% H_2_O_2_ for 10 min to block endogenous peroxidase and then with 10% normal goat serum for 10 min to block non-specific immunoglobulin binding. Subsequently, the sections were incubated at room temperature for 1 h with an antibody against MIG-7, rinsed and incubated for 30 min with a biotinylated secondary antibody followed by streptavidin-conjugated horse-radish peroxidase (HPR) for 30 min. The sections were treated with 3,3′-diaminobenzidine tetrahydrochloride (DAB) (Dako, Carpinteria, CA, USA) in 0.01% H_2_O_2_ for 10 min to visualize the reaction. Then, the slides were counterstained with Meyer's hematoxylin for 45 s and mounted. Finally, IHC was performed using an IHC kit (Jingmei Biotech Inc., Shanghai, China). Negative control slides were stained with rabbit serum. Evaluation of MIG-7 expression was simultaneously performed by 3 independent pathologists blinded about the clinicopathological features of the patients. The intensity of staining and the percentage of positive cells were used to create a composite score. The intensity of staining was scored as: 0 = none; 1 ≤ 10%, 2 = 10–50%; 3 ≥ 50%. The percentage of positive cells was scored as: 0 = no staining or weak staining (light yellow); 1 = moderate staining (yellow brown); 2 = strong staining (brown). MIG-7 expression score ranges from 0 to 4. Cases with score of 3 and 4 were considered to have high MIG-7 expression.

### Immunofluorescence

Equal number of SKOV3 cells with or without MIG-7 knockdown was seeded into a 12-well cell culture plate. Each well was placed a 0.2% gelatin pre-coated cell culture coverslip. The cells were fixed with 4% PFA and permeabilized with 0.2% Triton X-100, washed with PBS 3 times, blocked with 4% horse serum for 1 h at room temperature, and incubated with primary antibodies in blocking buffer overnight at 4°C. The cells were then washed with PBS 3 times and incubated with fluorochrome-conjugated secondary antibodies for 1 h. Cells were counterstained with DAPI, mounted with fluoromount G, and imaged using a fluorescent microscope (Zeiss, Jena, Germany) to generate pseudo color composite images.

### Wound healing and transwell migration assays

Confluent SKOV3 cells were scratched with 200 ml sterile tips in the center of the well horizontally and vertically to create gaps. After scratching, the cells were washed with sterile PBS to remove debris and cultured in fresh media. The gaps were imaged immediately and after 14 h. For transwell migration assay, 2×10^4^ SKOV3 cells were seeded in the lower chamber and 1×10^4^ human umbilical vein endothelial cells (HUVECs) were seeded in the top chamber. 100 ng/ml recombinant human VEGFA_165_ (R&D Systems, Minneapolis, MN, USA) was added to the bottom chamber that contained *MIG7*-knockdown SKOV3 cells. After 14h, HUVECs in the top chamber were removed by a cotton swab. HUVECs that migrated to the bottom chamber were stained with hematoxylin and enumerated under a microscope [19].

### MTT cell viability assay

5 000 SKOV3 or A2780 cells were seeded into each well of a 96-well plate in four replicates. The cells were incubated with various concentrations of D-39 for 14 h, washed and incubated in fresh medium containing 1 mg/ml 3-(4,5-dimethylthiazol-2-yl)-2,5-diphenyltetrazolium bromide (MTT) (Sigma-Aldrich, St. Louis, MO, USA), for another 4 h at 37°C. The medium was removed, and 150 ml MTT solvent (0.1% Triton X-100 and 4 mM HCl in isopropanol) was added to each well for incubation for 15 min. The optical density was determined at 570 nm [20].

### Clonogenic assay

3 000 SKOV3 cells seeded into 10-cm culture dishes were either left untreated or treated with 20 mM D-39 for 24 h. The medium was removed and cells were fixed with 2% paraformaldehyde for 30 min. The cells were gently washed with PBS twice and stained with hematoxylin for 5 min. After washing with PBS 3 times, the plates were dried and cell colonies were imaged and enumerated [21].

### Murine tumor xenograft model

Eight-week old 20 g female athymic nude mice of the C56BL/6 background were purchased from Shanghai Laboratory Animal Center and housed in the specific pathogen-free (SPF) facility of China Pharmaceutical University. The mice were inoculated with 10^6^ SKOV3 cells subcutaneously on the back. Tumor growth was monitored for 8 weeks, with tumor volume measured by external caliper every week. Mice were sacrificed after 60 days and tumor weight was determined. For the assessment of host survival, 10^7^ SKOV3 cells were subcutaneously inoculated into 8-week old female athymic nude mice of the C56BL/6 background. Mouse survival was monitored for up to 150 days. All animal procedures were approved by the Institutional Animal Care and Use Committee of China Pharmaceutical University.

### Chemical library screen

A library of 10^5^ compounds extracted from the medicinal plant *Liriope muscari* (Decne.) Baily was applied for inhibitor screening. Tianjin Tasly Medicine Distribution Group holds the patent of the compounds. 5×10^3^ SKOV3 cells were seeded in 384-well plates in a total volume of 40μl per well. On the following day, 0.8μl of 1mM compounds (in DMSO) was robotically pinned into each assay well to achieve a final concentration of 20 μM. An equal amount of DMSO was used as the vehicle control. After 24 hours of incubation, the culture medium was removed, and the cells were washed with cold PBS once. Each well of cells was directly lysed by adding 20 μl lysis buffer from a BIOG^HSC^ Super Probe One-Step PCR kit (BAIDAI, Changzhou, Jiangsu, China) and incubated at room temperature for 20 min. Another 2 μl stopping buffer from the kit was added to each well. After 2 min of incubation at room temperature, 15 μl of crude lysate from each well was used for reverse transcription using the kit according to manufacturer's instruction, followed by quantitative real-time PCR using SYBR Green mastermix (Thermo Fisher Scientific, Waltham, MA, USA) on an Applied Biosystems 7900 HT Fast Real-Time PCR System. The RT-PCR was performed at 95°C for 10 min followed by 95°C for 15 s and 60°C for 1 min for 60 cycles using primers specific for *MIG7* or *ACTB*. The compounds that gave ≥50% inhibition of *MIG7* transcript level were selected for further validation.

### Patient follow-up

Follow-up was performed at 3-month intervals after primary cytoreductive surgery. Physical examination was performed at every 3 months, which included serum cancer antigen 125 (CA-125) level, pelvic magnetic resonance imaging, color Doppler ultrasound of liver and kidney. X-ray was performed every 6 months.

### Statistical analysis

Statistical analysis was performed with SAS 9.2. *p* < 0.05 was considered significant. Qualitative variables were analyzed with the Pearson χ-square test and quantitative variables with Student's *t*-test. Overall survival (OS) of patients was defined by the interval from the date of primary cytoreductive surgery to the date of death, regardless of the cause or the last follow-up appointment. Progression-free survival was defined as the period after the conclusion of treatment to proven local recurrence or distant metastasis. The survivors were censored at the date of last contact. Logistic regression analysis and receiver operating characteristic curves were used to determine the predictive value of MIG-7. The area under the curve and the 95% confidence interval were used to assess the discriminatory power of MIG-7 for predicting prognosis. Kaplan-Meier analysis was used to determine the OS and progression-free survival (PFS). The Log-rank test was used to compare survival outcomes between patients and controls. The Cox-regression model was used to perform multivariate analysis to identify clinicopathological factors that independently predicted survival.

## DISCUSSION

The expression of MIG-7 is very low or undetectable in normal non-cancerous tissues, but significantly increased in a variety of cancer tissues, such as breast, lung, colon and endometrial cancer [14]. Most studies on the function of MIG-7 on tumorigenesis were focused on tumor invasion and metastasis. Recently, several reports suggested a correlation between MIG-7 overexpression and metastasis of lung cancer, possibly via the activation of the COX-2-PDE2 pathway and the induction of E-cadherin suppressors to promote epithelial-mesenchymal transition [13, 22, 23]. However, the function of MIG-7 in the pathogenesis of EOC and its association with clinicopathological parameters and its prognosis were unknown. We showed elevated expression of MIG-7 in EOC tissues but not normal ovary or ovarian cyst, which supports MIG-7 as a biomarker for EOC progression. MIG-7 expression negatively correlated with histopathological differentiation and lymph node metastasis, and positively correlated with FIGO stage, indicating that MIG-7 predicts poor prognosis of EOC. In addition, MIG-7 was important in promoting EOC pathogenesis. Knocking down *MIG7* impaired the proliferation, invasiveness and VEGFA expression of SKOV3 cells, and retarded tumor growth, reduced the formation of tumor-associated blood vessels *in vivo* and improved host survival after these cells were grafted. These functions of MIG-7 may underlie our clinical observation of the strong association between high MIG-7 expression and lymph node metastasis, poor histopathological differentiation of tumors and reduced survival of EOC patients.

Angiogenesis is central to both normal ovarian function and the development and progression of ovarian cancer [24, 25]. The mechanism underlying the proposed function of MIG-7 in vascular mimicry of several solid tumors was unknown [14, 26]. We revealed that, in EOC, this process likely encompasses the maintenance of the expression of VEGFA, a principal promoting factor of angiogenesis and metastasis. A number of angiogenesis-targeting therapies have been under clinical trial to treat EOC [27–31]. Most of them involved inhibition of the VEGF pathway by monoclonal antibodies, tyrosine kinase inhibitors or soluble decoy receptors. As MIG-7 is not expressed in normal ovarian tissues and is only detected during ovarian cancer development, MIG-7-targeted therapies should allow the specific inhibition of pathological angiogenesis associated with ovarian cancer development but spare physiological angiogenesis required for normal ovarian function. Our discovery of the MIG-7–VEGFA axis in EOC cells has additional preclinical and clinical implications for the treatment of EOC. EOC tends to give rise to abnormal vasculature characterized by leaky and immature vessels that are conducive to cancer cell metastasis [32], and high levels of VEGFA can transform normal, functional ovarian epithelium into ascites-producing cancers by enhancing vascular permeability [24, 33–36]. Indeed, there was a strong positive correlation between MIG-7 level in EOC tissues and ascites volume and lymph node metastasis in our cohort of patients. Thus, inhibiting MIG-7 in EOC not only may help to control tumor growth and angiogenesis, but also might reduce ascites formation and inhibit metastasis.

Consistent with the important function of MIG-7 in EOC development, our study found that MIG-7 expression was very low in normal ovarian tissues and ovarian cyst, but markedly elevated in EOC, thereby supporting MIG-7 as a potential biomarker for EOC. MIG-7 expression negatively correlated with histopathological differentiation and positively correlated with FIGO stage, indicating that high MIG-7 level predicts poor prognosis of EOC. Notably, EOC patients with a high level of MIG-7 were usually accompanied with a high percentage of lymph node metastasis, which might be explained by the function of MIG-7 in promoting the migration of EOC cells found in our *in vitro* study. Furthermore, patient follow-up study revealed that high ovarian MIG-7 level had a significant negative impact on 3 and 5-year OS of EOC patients following primary cytoreductive surgery. In a Poisson multivariate analysis, MIG-7 level and lymph node metastasis were the only two parameters among other major clinicopathological parameters including residual tumor size, FIGO stage, ascites volume, histopathological differentiation and serum CA-125 level, a widely used biomarker for OC diagnosis [37], that significantly associated with reduced OS. Therefore, MIG-7 may represent a reliable and independent indicator of EOC prognosis.

The potential therapeutic efficacy of inhibiting MIG-7 in EOC is further corroborated by the ability of the small molecule inhibitor D-39 from the medicinal plant *L. muscari* (Decne.) Baily to suppress the growth, migration and VEGFA expression of SKOV3 cells. Of note, D-39 exhibited specific inhibition of the expression of MIG-7 but not other MIG family members and selective killing of MIG-7-expressing SKOV3 cells. The specificity of D-39 on suppressing VEGFA over a variety of other angiogenic and growth factors echoed the requirement for MIG-7 in maintaining VEGFA expression. The mechanism underlying such potent and specific inhibition of MIG-7 expression by D-39 is under investigation.

In summary, we have demonstrated MIG-7 as an important promoting factor of multiple key processes of EOC pathogenesis, and identified MIG-7 as a novel prognostic biomarker of EOC. Our findings provide a strong basis for the therapeutic inhibition of MIG-7, such as using the small molecule inhibitor D-39 or MIG-7-targeting monoclonal antibodies [12], to treat EOC.

## SUPPLEMENTARY FIGURES AND TABLES


