# Supplementary material for: Migration-inducing gene 7 promotes tumorigenesis and angiogenesis and independently predicts poor prognosis of epithelial ovarian cancer
Source: Oncotarget. 2016 Mar 30;7(19):27552–66. doi: 10.18632/oncotarget.8487 (PMC5053671; doi:10.18632/oncotarget.8487)
Supplement: Supplementary file 1 [file oncotarget-07-27552-s001.pdf]

SUPPLEMENTARY FIGURES AND TABLES

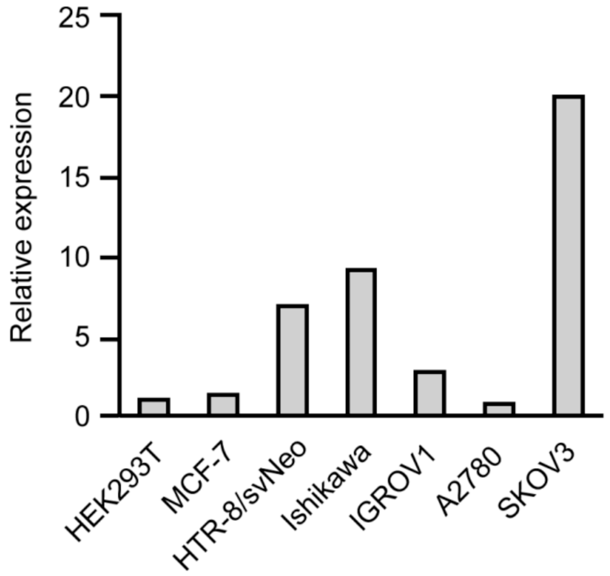

Supplementary Figure S1: SKOV3 cells exhibit high *MIG7* expression among several human epithelial cancer cell lines. qRT-PCR analysis of *MIG7* expression in the breast adenocarcinoma line MCF-7, extravillous trophoblast cell line HTR-8/svNeo, endometrial adenocarcinoma line Ishikawa, ovarian carcinoma lines IGROV1, A2780 and SKOV3. The values were normalized to *MIG7* expression in the human embryonic kidney cell line HEK293T, which was assigned to be 1. The data represent the mean of the triplicates of each cell line.

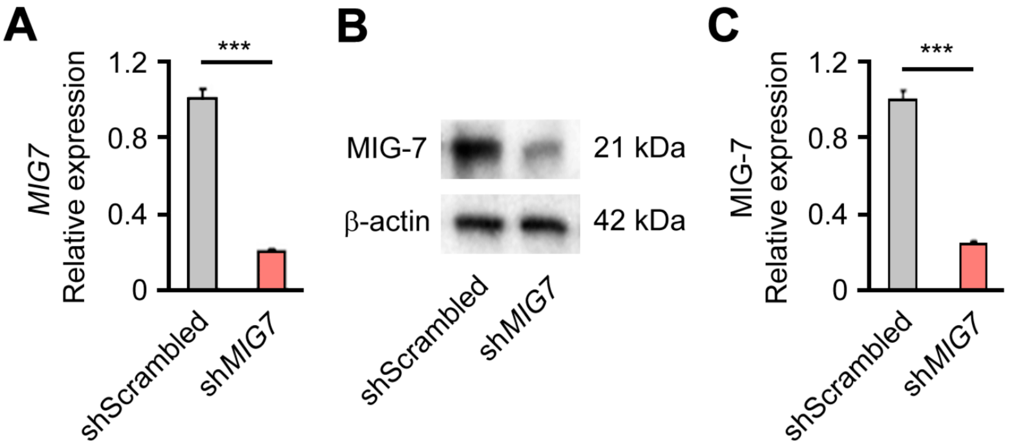

Supplementary Figure S2: Reduced expression of MIG-7 in SKOV3 cells after stable knockdown. A. qRT-PCR analysis of *MIG7* gene expression in SKOV3 cells after stable knockdown with scrambled or *MIG7*-targeting shRNA. **B, C.** Western Blot and densitometric analyses of MIG-7 protein levels in SKOV3 cells after stable knockdown. β-actin served as a loading control. Data are presented as mean ± SEM of triplicates, and represent 3 independent experiments.***: $p < 0.001$. $n = 3$.

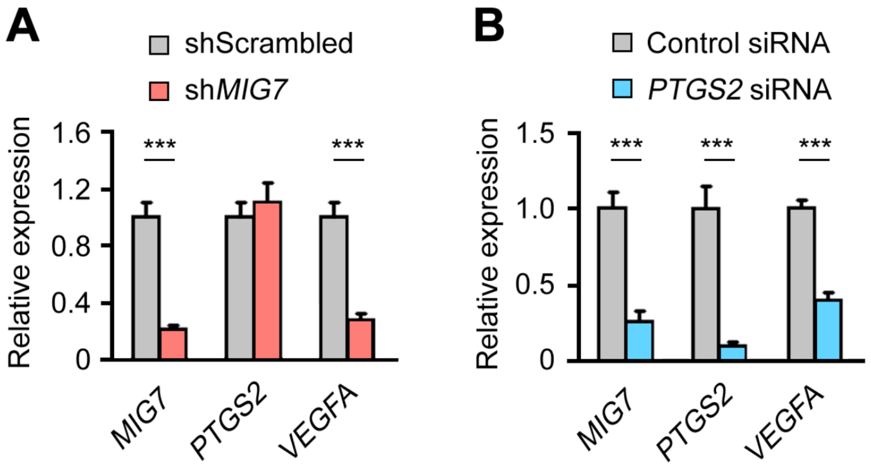

Supplementary Figure S3: MIG-7 is required for VEGFA expression in SKOV3 cells. A. qRT-PCR analysis of *MIG7*, *PTGS2* (encoding COX-2) and *VEGFA* expression in SKOV3 with control or *MIG7* stable knockdown. **B.** qRT-PCR analysis of *MIG7*, *PTGS2* and *VEGFA* expression in SKOV3 cells with control or *PTGS2* stable knockdown. Data are presented as mean ± SEM of triplicates and represent 3 independent experiments. ***: $p < 0.001$. $n = 3$.

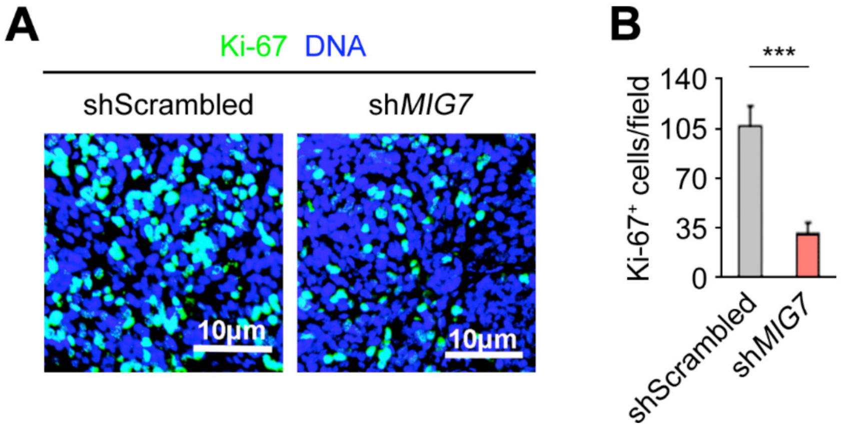

Supplementary Figure S4: MIG-7 promotes proliferation of EOC cells *in vivo*. A, B. Immunofluorescent images and statistical analysis of Ki-67 (green) expression in resected tumor tissues of athymic nude mice 60 days after subcutaneous inoculation of SKOV3 cells with control or *MIG7* stable knockdown. DAPI stained DNA (blue). Data are presented as mean ± SEM of the tumors resected from 5 mice per group, and represent 3 independent experiments. Bar: 10 mm. ***: $p < 0.001$. $n = 5$.

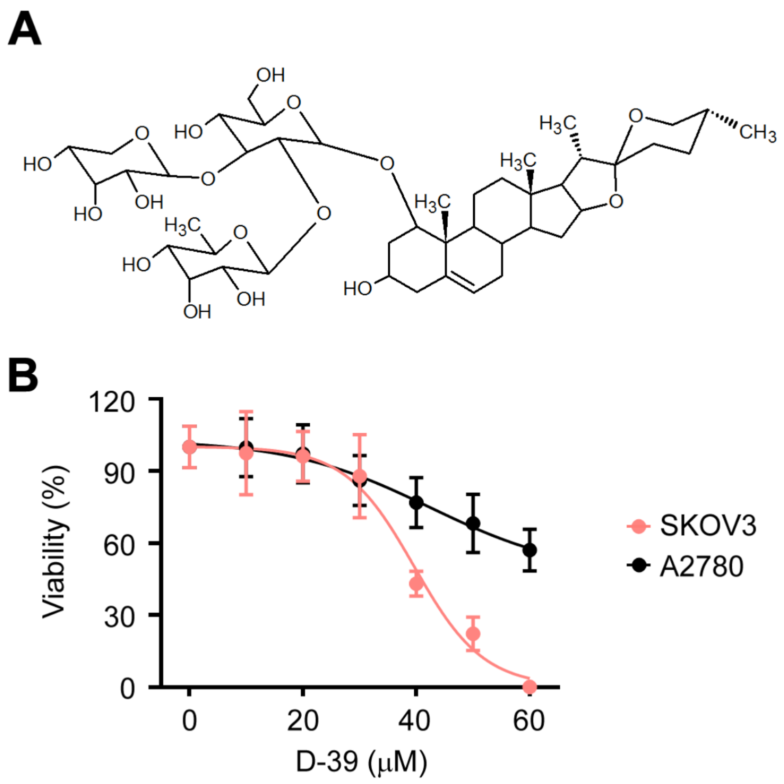

Supplementary Figure S5: The inhibitor D-39 exhibits selective killing of MIG-7-expressing SKOV3 cells. A. Chemical structure of the inhibitor D-39. **B.** Proliferation of SKOV3 and A2780 cells after 16 h of treatment with D-39, as determined by an MTT assay. Data are presented as mean ± SEM of 4 replicates, and represent 3 independent experiments. The percentage of viable cells was normalized to untreated cells.

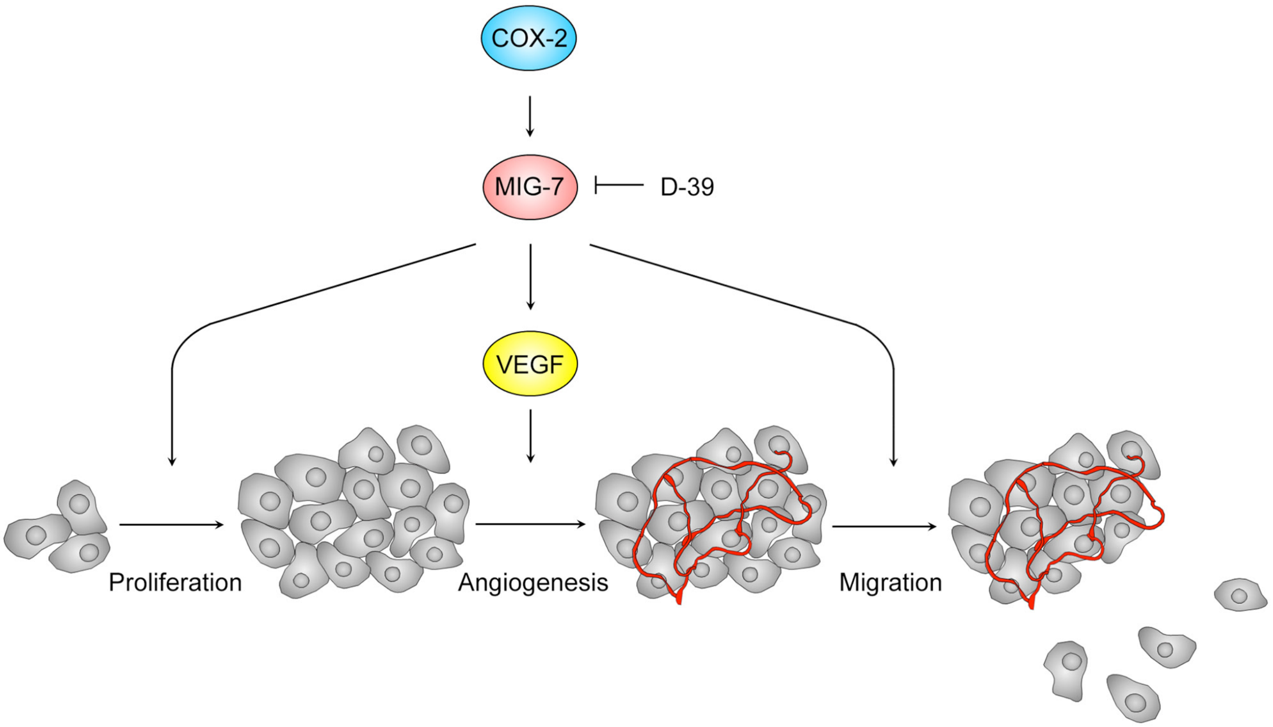

Supplementary Figure S6: A proposed model of MIG-7-mediated regulation of EOC growth and angiogenesis. Elevated MIG-7 in EOC cells upregulates the expression of VEGFA, there by promoting tumor growth and angiogenesis. In addition, MIG-7 enhances the proliferation and invasion of EOC cells. Collectively, these effects promote EOC angiogenesis, growth and metastasis. The inhibitor D-39 blocks the expression of MIG-7 and suppresses these multiple critical steps in EOC pathogenesis.

Supplementary Table S1: shRNA and siRNA used in the study

Target Gene		Sequence
MIG7 shRNA	Sense	5′- CACCGCAAGTACAGGGCAGAATTTCTTCA AGAGAGAAATTCTGCCCTGTACTTGCTTTTTTG-3′
	Anti-sense	5′- GATCCAAAAAAGCAAGTACAGGGCAGAATT TCTCTCTTGAAGAAATTCTGCCCTGTACTTGC-3′
PTGS2 siRNA		5′- AACACCGGAATTTTTGACAAG-3′

Supplementary Table S2: Primers used in qRT-PCR analysis

Gene		Sequence
ACTB	Sense	5′-CATGTACGTTGCTATCCAGGC-3′
	Anti-sense	5′-CTCCTTAATGTCACGCACGAT-3′
FGF2	Sense	5′-AGTGTGTGCTAACCGTTACCT-3′
	Anti-sense	5′-ACTGCCCAGTTCGTTTCAGTG-3′
HGF	Sense	5′-GCTATCGGGGTAAAGACCTACA-3′
	Anti-sense	5′-CGTAGCGTACCTCTGGATTGC-3′
IGF1	Sense	5′-GCTCTTCAGTTCGTGTGTGGA-3′
	Anti-sense	5′-GCCTCCTTAGATCACAGCTCC-3′
MIG2	Sense	5′-TGTGGAGCAGATCAATCGCAA-3′
	Anti-sense	5′-GTTGGGCAACCGAAGGATGA-3′
MIG6	Sense	5′-CTGGAGCAGTCGCAGTGAG-3′
	Anti-sense	5′-GCCATTCATCGGAGCAGATTTG-3′
MIG7	Sense	5′-CACCTGCCTCTGGTCGTTAGG-3′
	Anti-sense	5′-TACTGGATTCCTCTAGCTTTGGTGTT-3′
MIG14	Sense	5′-ATGAGGGCCGTTACTATGAATGT-3′
	Anti-sense	5′-CCTTGGTGAAGCCTCCATTTTG-3′
PDGFA	Sense	5′-GCAAGACCAGGACGGTCATTT-3′
	Anti-sense	5′-GGCACTTGACACTGCTCGT-3′
PDGFB	Sense	5′-CTCGATCCGCTCCTTTGATGA-3′
	Anti-sense	5′-CGTTGGTGCGGTCTATGAG-3′
PGF	Sense	5′-GAACGGCTCGTCAGAGGTG-3′
	Anti-sense	5′-ACAGTGCAGATTCTCATCGCC-3′
PTGS2	Sense	5′-CTGGCGCTCAGCCATACAG-3′
	Anti-sense	5′-CGCACTTATACTGGTCAAATCCC-3′
TGFB1	Sense	5′-CTAATGGTGGAAACCCACAACG-3′
	Anti-sense	5′-TATCGCCAGGAATTGTTGCTG-3′
TNF	Sense	5′-CCTCTCTCTAATCAGCCCTCTG-3′
	Anti-sense	5′-GAGGACCTGGGAGTAGATGAG-3′
VEGFA	Sense	5′-AGGGCAGAATCATCACGAAGT-3′
	Anti-sense	5′-AGGGTCTCGATTGGATGGCA-3′
VEGFB	Sense	5′-GAGATGTCCCTGGAAGAACACA-3′
	Anti-sense	5′-GAGTGGGATGGGTGATGTCAG-3′
VEGFC	Sense	5′-GGCTGGCAACATAACAGAGAA-3′
	Anti-sense	5′-CCCCACATCTATACACACCTCC-3′

Supplemental Table S3: Antibodies used in this study

Antigen	Manufacturer	Cat. No	Host species	Application and Dilution
β-actin	Abcam	ab5694	Rabbit	WB (1:1000)
CD31	Abcam	ab28364	Rabbit	IF and IHC (1:100)
HSP90	BD Biosciences	610418	Mouse	WB (1:1,000)
Ki-67	Cell Signaling	9129	Rabbit	IF (1:200)
MIG-7	Abcam	ab83494	Rabbit	IHC (1:100); WB (1:500)
VEGF	Abcam	ab46154	Rabbit	IF and IHC (1:100);WB (1:500)

IF: Immunofluorescence
IHC: Immunohistochemistry
WB: Western Blot
